# Acetylcholinesterase and butyrylcholinesterase in cardiosurgical patients with postoperative delirium

**DOI:** 10.1186/s40560-017-0224-1

**Published:** 2017-05-26

**Authors:** Mira John, E. Wesley Ely, Dorothee Halfkann, Julika Schoen, Beate Sedemund-Adib, Stefan Klotz, Finn Radtke, Sebastian Stehr, Michael Hueppe

**Affiliations:** 1 Clinic for Anaesthesiology and Intensive-Care Medicine, UKSH Campus Luebeck, Ratzeburger Allee 160, 23538 Luebeck, Germany; 2 Pulmonary and Critical Care Medicine, Vanderbilt University, Nashville, Tennessee USA; 3 Geriatric Research Education Clinical Center (GRECC) of the Tennessee Valley Veterans Administration, Nashville, Tennessee USA; 4 Department of Cardiac and Thoracic Vascular Surgery, UKSH Campus Luebeck, Luebeck, Germany; 5 Clinic for Anaesthesiology and Operative Intensive-Care Medicine, Charité University Hospital Berlin, Berlin, Germany

**Keywords:** Delirium, Cardiac surgery, Intensive care unit, Acetylcholinesterase, Butyrylcholinesterase, CAM-ICU, Nu-DESC

## Abstract

**Background:**

Patients in intensive care units (ICU) are often diagnosed with postoperative delirium; the duration of which has a relevant negative impact on various clinical outcomes. Recent research found a potentially important role of acetylcholinesterase (AChE) and butyrylcholinesterase (BChE) in delirium of critically ill patients on non-surgical ICU or in non-cardiac-surgery patients. We tested the hypothesis that AChE and BChE have an impact on patients after cardiac surgery with postoperative delirium.

**Methods:**

After obtaining approval from the local ethics committee, this mechanistic study gathered data of all 217 patients included in a randomized controlled trial testing non-pharmacological modifications of care in the cardiac surgical ICU to reduce delirium.

Delirium was assessed with the Confusion Assessment Method for the Intensive Care Unit (*CAM-ICU*) and the Nursing Delirium Screening Scale (*Nu-DESC*) twice a day for the first 3 days after surgery. Further outcome variables were somatic laboratory parameters and variables regarding surgery, anesthesia, and postsurgical recovery. 10 μl venous or arterial blood was drawn and AChE and BChE were determined with *ChE check mobile* from Securetec.

**Results:**

Of 217 patients, 60 (27.6%) developed postsurgical delirium (POD). Patients with POD were older (*p* = 0.005), had anemia (*p* = 0.01), and worse kidney function (*p* = 0.006). Furthermore, these patients had lower intraoperative cerebral saturation (NIRS) (*p* < 0.001) and higher intraoperative need of catecholamines (*p* = 0.03). Delirious patients showed more inflammatory response (*p* < 0.001). AChE and BChE values were mainly inside the norm. Patients with values outside the norm did not have POD more often than others. Regarding AChE and BChE patients did not differ in having delirium or not (*p* > 0.10).

**Conclusions:**

Postoperative measurement of AChE and BChE did not discern between patients with and without POD. The effect of the cardiac surgical procedure on AChE and BChE remains unclear. Further studies with patients in cardiac surgery are needed to evaluate a possible combination of delirium and the cholinergic transmitter system. There might be possible interactions with AChE/BChE and blood products and the use of cardiopulmonary bypass, which should be investigated more intensively.

**Trial registration:**

German Clinical Trials Register, DRKS00006217

## Background

The definition of delirium according to DSM-5 criteria includes a transient and serious disturbance in attention and cognition and the development over a short period of time. The symptoms tend to fluctuate during the day and cannot be explained by a pre-existing neurocognitive disorder [1].

Delirium is a complex symptom which is very common in operative and non-operative disciplines in the course of hospital stay. The incidence is especially high among patients undergoing heart surgery [2]. The incidence in this patient population has been described to be from 30 up to 80% [2–4].

The duration of delirium has a relevant negative impact on various clinical outcomes. Patients stay longer on the ICU, they suffer from more complications, are immobilized for a longer period of time, have a higher 6-months-mortality, and have long-term cognitive impairment [3–8].

The causes for delirium are numerous. Intoxication, drugs, alcohol, hypoglycaemia, hypoxia, or anemia might lead to cognitive dysfunction. The pre-, intra-, and postoperative status of patients might also trigger symptoms of delirium. Especially, a pre-existing dementia, fluid loss, abnormal electrolyte concentrations, use of benzodiazepines, and hypotension belong to those trigger factors. Especially older patients tend to develop delirium more often. This might be a result of hearing impairment, changes of the environment, and a limited bladder- and bowl function; in particular, during stay in hospital [9].

Other hypotheses assume an influence of infection and stress (also postoperative stress) with a higher level of cytokines (IL-1, IL-6) and a higher level of cortisol inside the cerebrospinal fluid [10, 11].

In addition, neuronal metabolism and changes in transmitter interaction have been implicated to play a central role in delirium (e.g., acetylcholine and dopamine) [12, 13]. Previous studies show that patients with preoperative lower levels of cholinesterase are postoperatively more often diagnosed with delirium [14]. Those patients might have a higher function of serum-cholinesterase. Additionally, pilot studies show that the use of indirect parasympathomimetics (e.g., physostigmine) may reduce the severity of delirium [15–17]. Worek et al. found a procedure which provides a simple method for sensitive and precise determination of AChE in whole blood samples [18].

Due to recent studies that found a potentially important mechanistic role of acetylcholinesterase (AChE) and butyrylcholinesterase (BChE) in delirium of critically ill patients on non-surgical ICU or in non-cardiac surgery patients, the main focus of this paper was to examine AChE and BChE in patients after cardiac surgery and their association with postoperative delirium [12, 14, 16, 19].

## Methods

### Study design

We designed a mechanistic study of data from all patients included in a randomized controlled trial. The two investigators were blinded against each other. The study was conducted after the positive vote of the local ethics committee and was registered in the German Register for Clinical Trials (German Clinical Trials Register: DRKS00006217). All patients gave informed consent to take part in this study.

### Patient population

Patients were enrolled from June 1st, 2014 until December 20th, 2014. All patients had an elective cardiosurgical procedure with a postoperative stay on the ICU.

Inclusion criteria: minimum age of 18 years, men and women were equally considered, command of the German language.

Exclusion criteria: missing consent, preoperative diagnosis of delirium (with *CAM-ICU*), psychiatric pre-diagnosis of schizophrenia, preoperative indications for cognitive dysfunction (*abbreviated mental test* < 7 points), *RASS-Score* < −2 for the current test, and neurological complications (e.g., media infarction).

### Independent variable

The intervention itself was the study’s independent variable and the assignment to control- or intervention group. Patients in the intervention group received special exercises for orientation: standardized acoustic, visual, olfactory, and tactile stimulation (see Table 1). The intervention was performed twice a day for the first 3 days after surgery.

**Table 1:** Exercises for orientation

Stimulation	Contents	Summary
Acoustic/visual stimulation	Time of year, date, time of day, weather, place, reason for stay, day of surgery/duration of stay News of the day (newspaper article with picture)	Summary with pictures of temporal and local orientation
Olfactory stimulation	Lemon Peppermint Orange	Summary with plants suitable to the smelling and view at the items of tactile stimulation
Tactile stimulation	Screw Cotton pad Sandpaper	

Annotation*:* acoustic stimulation contained questions for the time of year, the date and time of day as well as the weather, place, reason, and duration of stay. Patients were corrected in case of false answer or supported in case of correct answers. The information was summarized by the investigator and was enhanced with suitable pictures. Afterwards patients got to read out a newspaper article. In the olfactory stimulation, patients got to smell three different smellings (lemon, peppermint, and orange).

The answers were corrected or supported and summarized with special pictures. The tactile stimulation in the end included a screw, cotton pad, and a sandpaper. The answers were corrected or supported, and patients could have a look at the items they felt before

### Dependent variable

#### Delirium

Occurrence of postsurgical delirium (POD) was the dependent variable (i.e., the primary outcome variable), as measured using a combination of the Confusion Assessment Method for the Intensive Care Unit (CAM-ICU) [20] and the Nursing Delirium Screening Scale (Nu-DESC) [21, 22] twice a day for the first 3 days after surgery (morning and late afternoon). Both screening tools were tested for ICU patients and show high sensitivity and specificity.

CAM-ICU was performed identically each time and inspired by the CAM-ICU worksheet. All four features were conducted (acute onset or fluctuating course, inattention, altered level of consciousness, disorganized thinking); the inattention was tested via the letters in the word ANANASBAUM, explained once at the beginning and in case of patients failing to squeeze on the letter “A” and when the patients squeezed on any letter other than “A” errors were counted.

Patients with a Richmond Agitation Sedation Scale (RASS) ≤ −2 were excluded for the current testing. A positive delirium diagnoses was given if patients got a positive result in CAM-ICU and/or Nu-DESC at least once within the 3 days of measurement.

#### Cognitive dysfunction

For recognition of cognitive dysfunction, the abbreviated mental test (AMT) was performed once a day (in the evening) for the first 3 days after surgery. Especially for cardiosurgical patients the AMT is a very useful tool in detection for cognitive function.

#### Anxiety and pain

According to the current DAS-Guidelines (Guideline for **D**elirium management, **A**nalgesia, **S**edative medication on the ICU), patients were interviewed for anxiety and pain also twice a day for the first 3 days after surgery. It was assessed with the numeric rating scale (NRS; score*:* 0 = no pain–10 = maximum pain). Pain was separated into resting pain and pain on movement.

#### Somatic laboratory parameters

Blood was drawn from each patient twice a day after each testing. All patients had a central venous catheter or an arterial catheter where 10 μl venous or arterial blood was taken out and used for determination of AChE and BChE. The measurement was performed immediately with *ChE check mobile* from Securetec [18]. The reference range is for AChE: 26.7–50.9 U/gHb and for BChE 2300–7000 U/L.

Furthermore, postoperative leukocytes, C-reactive protein (CRP), creatine kinase (CK), heart-enzymes (CK-MB), and creatinine were tested once every day in the normal laboratory control.

### Preoperative variables

Patients were evaluated for psychological and cognitive function. After a written consent, patients received a 12-sided questionnaire with special questions about personal, the Hospital Anxiety and Depression Scale in a German version (HADS-D), the Pain Sensitivity Questionnaire (PSQ), and finally questions for life quality (SF-12). Questions about the person itself contained information about age, gender, education, previous surgery, regular use of nicotine and alcohol, other illnesses, and use of drugs.

Preoperative assessment of CRP, leukocytes, hemoglobin, and creatinine was performed.

### Variables of surgery and anesthesia

From the anesthesia report duration of surgery, method of narcosis and its duration as well as intraoperative cerebral saturation (near infra-red spectrometry, NIRS), lactate, hemoglobin, and the used drugs were noticed. Furthermore, transfusion of blood products, the use of catecholamines, and any complications were written down.

### Postoperative variables

Especially important was the duration of mechanical ventilation and the time of stay on the ICU and in hospital in total. The need of blood transfusion, given psychiatric drugs, and any other medication was collected. Furthermore, any complication in recovery time was noticed.

### Study protocol

All elective cardiosurgical patients were assessed for eligibility. In case of meeting the inclusion criteria, a written consent from patients were evaluated and randomized. The randomization was performed by the project leader (M.H.) who was not involved in the delirium assessment and the implementation of the intervention. The program BiAS was used for randomization.

All patients were postoperatively admitted to the ICU mechanically ventilated and hemodynamic supported. As soon as patients met the extubation criteria and were extubated, both investigators visited the patients separately (blinded by each other) twice a day (in the morning and in the late afternoon) for the first 3 days after surgery. The intervention, delirium assessment, and the postoperative evaluation were performed each time of measurement.

### Statistical analysis

Statistical analysis was performed by chi-square tests, *t* tests, and analysis of variance using SPSS 22. *P values* lower 0.05 were considered statistically significant.

## Results

### Sample of analysis

**Fig. 1:**
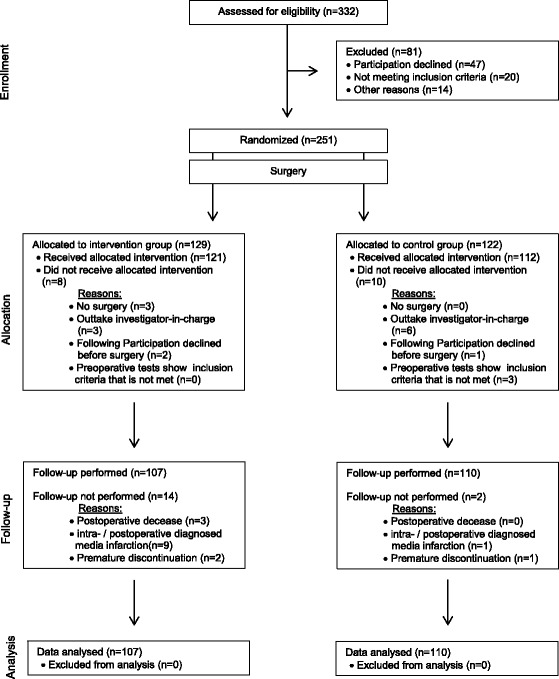
Flowchart (Consort 2010)

332 patients were assessed for eligibility. All those patients had an elective cardiosurgical procedure. Eighty-one patients had to be excluded due to declining participation (*n* = 47), missing inclusion criteria (*n* = 20), or others (*n* = 14). In total, 251 patients were randomized and allocated to intervention (*n* = 129) or control group (*n* = 122). Due to canceled surgery (*n* = 3) or later decline of participation (*n* = 6), preoperative tests that revealed exclusion criteria (*n* = 3), postoperative decease of patients (*n* = 3), diagnosed media infarction (*n* = 10), or others (*n* = 9), a total number of 217 patients were included in the analysis (see Fig. 1). Within this mechanistic study in up to six patients few data was missing.

### Description of sample of analysis

Most participants were male gender (71.4%), had a lower education and any previous surgeries. Patients were classified as ASA-PS III in 92.6% (patients with illnesses with marked impairment). All patients received a balanced anesthesia with propofol, sufentanil, remifentanil and sevoflurane. Most patients had coronary surgery (40.6%). The mean duration of the anesthetic procedure was 5.5 h, surgery lasted about 4.75 h. Cardiac bypass time was 2 h in average. Patients were sedated and ventilated for about 3.5 h in postoperative ICU care. All patients got opiates for analgesia. In case they developed delirium, they got lorazepam or haloperidol. If they could not be extubated early, they had a combined sedation with dexmedetomidine or propofol and opiates. In case the weaning lasted more than a week, propofol was replaced by midazolam. On average, patients were discharged to a normal ward after 2.1 days and left the hospital after 12.6 days. Table 2 gives an overview of sample of analysis description.

**Table 2:** Description of sample of analysis

Characteristic	Total sample (*n* = 217)
Age (years) [M (SD)]	65.4 (12.3)
Sex [*n* (%)]	
Male	155 (71.4)
Female	62 (28.6)
Education [*n* (%)]	
No graduation	7 (3.2)
Low graduation	89 (41.0)
Middle graduation	52 (24.0)
Polytechnic secondary school	28 (12.9)
Higher graduation	21 (9.7)
A-level	20 (9.2)
ASA-PS [n (%)]	
2	2 (0.9)
3	201 (92.6)
4	14 (6.5)
LV-EF in % [M (SD)]	57.9 (13.4)
Anesthesia [*n* (%)]	
Balanced	217 (100.0)
Pulmonary artery catheter	59 (28.5)
NIRS	217 (100.0)
TEE	169 (81.6)
BIS	217 (100.0)
Operative procedure [*n* (%)]	
Coronary surgery	88 (40.6)
Valve surgery (even multiple valves)	46 (21.2)
Coronary and valve surgery	22 (10.1)
Aorta ascendens replacement	2 (0.9)
Valve surgery + aorta ascendens replacement	12 (5.5)
Other combinations	35 (16.1)
Other surgery	12 (5.5)
Intraoperative variables [M (SD)]	
Duration of anesthesia (min)	329.2 (81.1)
Duration of surgery (min)	258.5 (79.1)
Time of bypass (min)	123.2 (58.9)
Duration of mechanical ventilation after surgery (min) [M (SD)]	334.5 (658.3)
Duration of stay on the ICU (days) [M (SD)]	2.1 (2.8)
Duration of stay in hospital (days) [M (SD)]	12.6 (6.2)

Abbreviation: *M* mean, *SD* standard deviation, *n* number of patients, *%* percent, *min* minutes. *ASA* risk score American Society of Anesthesiologists, *LVEF* left ventricular ejection fraction, *NIRS* near infrared spectrometry,*TEE* transesophageal echocardiography, *BIS* bispectral index)

### Incidence of delirium

Of 217 patients, 60 (27.6%) developed postoperative delirium according to the results of CAM-ICU and Nu-DESC combined together. Considering just the results from CAM-ICU, only 26 patients had a positive result (12.0%). There were no differences in transient and continuously delirious patients. Most patients developed their delirium on the second day after surgery.

### Differences between patients with and without postoperative delirium

#### Preoperative variables

Patients who developed postoperative delirium were older (*p* = 0.005). They did not differ in their education. Values of hemoglobin were already preoperatively lower in patients developing delirium afterwards (*p* = 0.01). Furthermore, those patients had worse kidney function (*p* = 0.006). We did not see a difference in preoperatively recognized higher inflammatory response regarding the delirium diagnosis. Patients with delirium did not have a higher ASA-PS-Score preoperatively than patients without delirium.

#### Intraoperative variables

If patients had a lower intraoperative cerebral saturation they developed delirium postoperatively more often (*p* = 0.001). Additionally, those patients had a higher need of noradrenaline (*p* = 0.03) during surgery. The duration of anesthesia, surgery or time of bypass was not longer in patients with postoperative delirium (*p* > 0.05). Furthermore, there was no relation in occurrence of complications (heart arrhythmia and intubation problems) and a higher rate of delirium afterwards.

#### Postoperative variables

Patients with POD had a longer duration of ventilation after surgery (*p* = 0.004) and increased length of ICU as well as in hospital stay (both *p* = 0.01). Neurological complications during hospital stay were seen more often in patients with delirium (*p* = 0.001). Anemia was also more often present in patients with POD than without (*p* = 0.01). Other complications were recorded more often in delirium patients, but without reaching the level of significance.

Patients with delirium did show lower value of hemoglobin (*p* = 0.002), higher inflammatory response (CRP: *p* = 0.01), higher rate of heart enzymes (CK: *p* = 0.03; CK-MB: *p* = 0.04), and worse kidney function (creatinine: *p* = 0.001). Leukocytes did not differ in patients with or without delirium.

Leukocytes and heart enzymes declined during the 3 days of measurement. CRP reached its maximum on the third day after surgery. Hemoglobin and creatinine remained stable. Table 3 shows values in detail.

**Table 3:** Pre-, intra-, and postoperative variables

Variables	Delirum	*n*	M	SD	*p* value
Preoperative variables					
Age	No	157	64.0	12.8	*0.005*
	Yes	60	69.2	10.1	
Hemoglobin (g/dl)	No	157	13.6	1.8	*0.01*
	Yes	60	13.0	1.8	
Creatinine (μmol/l)	No	155	93.3	39.5	*0.006*
	Yes	59	116.4	81.4	
Intraoperative variables					
Duration of anesthesia (min)	No	157	323.3	77.3	0.08
	Yes	60	344.7	89.1	
Duration of surgery (min)	No	151	252.4	74.3	0.07
	Yes	60	274.0	88.7	
Time of bypass (min)	No	152	119.9	57.2	0.35
	Yes	60	128.3	63.6	
NIRS (low)	No	157	69.5	8.2	*0.001*
	Yes	60	65.4	7.6	
Nor-adrenaline	No	157	2.9	2.8	*0.03*
	Yes	60	3.8	2.4	
Postoperative variables					
Mechanical ventilation (min)	No	157	254.4	178.8	*0.004*
	Yes	60	544.1	1200.2	
Stay on the ICU (days)	No	157	1.9	2.5	*0.01*
	Yes	60	2.9	3.4	
Stay in hospital (days)	No	157	11.9	5.3	*0.01*
	Yes	60	14.3	8.0	

Abbreviation: *M* mean, *SD* standard deviation, *n* number of patients, *min* minutes

*p* < 0.05 is statistically significant

#### AChE and BChE

**Fig. 2:**
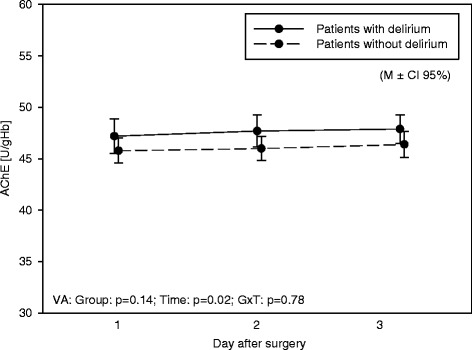
Acetylcholinesterase. AChE values over the three days of measurement in patients with and without delirium Abbreviation: variance of analysis: group, *Time* time, *G × T* group × time. *p* p - value, *M ± CI 95%* mean ± confidence interval 95%

**Fig. 3:**
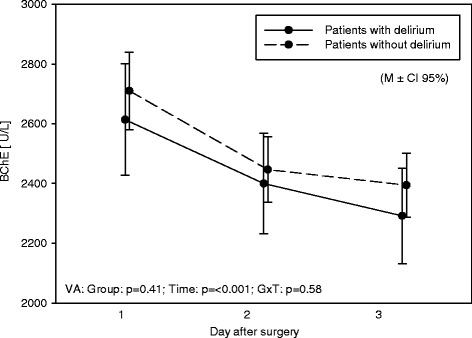
Butyrylcholinesterase. BChE values over the 3 days of measurement in patients with and without delirium Abbreviation: variance of analysis: group, *Time* time, *G × T* group × time. *p* p value, *M ± CI 95%* mean ± confidence interval 95%

AChE increased within the first 3 days after surgery (*p* = 0.02), BChE decreased (*p* < 0.001) (Table 4). Patients with and without delirium did not differ (Figs. 2 and 3).

**Table 4:** AChE and BChE values (in detail)

Variable	Day	Delirium		No delirium		Analysis of variance					
						Group		Time		G × T	
		M	SD	M	SD	F	*p*	F	*p*	F	*p*
AChE	1	47.2	5.8	45.8	6.2	2.2	0.14	4.3	0.02	0.2	0.78
	2	47.7	5.4	46.0	5.9						
	3	47.9	4.8	46.4	6.3						
BChE	1	2613.8	639.5	2710.2	632.2	0.7	0.41	62.7	<0.001	0.5	0.58
	2	2399.8	574.6	2446.3	534.0						
	3	2291.7	548.0	2394.2	522.4						

Abbreviation: *M* mean, SD standard deviation, *p* p - value, *G* × *T* group × time

Just a few values were outside the normal range; however, those patients did not develop delirium more often than others. Additionally, AChE and BChE levels were not affected by the study intervention.

#### Cognitive function

Patients with postoperative delirium did not have a worse AMT preoperatively in comparison to patients without delirium. Patients reached a worse result in AMT during an episode of postoperative delirium (*p* < 0.001).

#### Anxiety and pain

Patients with and without delirium did not differ in having resting pain and pain under movement. Patients showed significantly more anxiety when they developed delirium (*p* = 0.001).

## Discussion

### Summary of key findings

The focus of this analysis was a comparison of patients with and without delirium after cardiac surgery.

Within this patient population, the incidence of postoperative delirium was 27.6%. Secondary outcome variables showed that patients with delirium were older, had anemia, a worse kidney function, a lower cerebral saturation during surgery, and a higher need of noradrenaline. Furthermore, those patients had a higher inflammatory response.

The main result was that AChE and BChE values were mainly inside the norm and did not differ in patients having postoperative delirium or not.

Due to recent studies which supposed an important role of AChE and BChE in delirium of critically ill patients this study focused on AChE and BChE in patients after cardiac surgery and its impact on postoperative delirium. Results of this study did not show a difference in AChE and BChE of patients suffering from delirium than those who did not.

### Strengths and limitations

This analysis was designed as a mechanistic study from all patients included in a randomized controlled trial. The investigators were blinded against each other. Those two facts show the quality and strength of this study. Additionally, patients were visited twice a day which takes the cycling of delirious symptoms into account. Furthermore, most patients undergoing heart surgery participated and responded very well. The dependent variable (the occurrence of postoperative delirium) was assessed with CAM-ICU and Nu-DESC. Both screening tools are characterized by its good test quality criteria (sensitivity and specificity) and the easy use for critically ill patients on the ICU. Additionally, testing was performed by a qualified investigator who was independent of the work on the ICU. As shown in the section of results, this study could reproduce previous findings in delirious patients. The differences in patients with and without delirium are plausibly significant. As well as other studies have already shown patients with delirium were older, had more often anemia, were mechanically ventilated longer, had longer ICU and hospital stays; furthermore, as previously shown, those patients had a higher rate of cognitive dysfunction. These facts indicate the strength and validity of this analysis.

Limitations of this study might be the short duration of 3 days measurement, no preoperative values of AChE and BChE as well as no long-term follow-up was performed. Blood was taken from each patient; in case the analysis could not be performed immediately, the sample was cooled down in a refrigerator. Maybe values of AChE and BChE changed in combination with lower temperatures. During analysis, the blood needed to be mixed with a substance in the cap; maybe there is a difference in strength of shaking and final discrepancies of the mixed samples. Furthermore, it was only one measurement performed with each sample, so no control values could be achieved.

In addition, not all patients got a measurement due to technical problems (long wait for material) and removed central line catheters which implicated missing values afterwards.

Previous studies assumed an interaction of delirium and the immune and cholinergic systems [12] and identified plasma cholinesterase activity as a useful biomarker to identify patients with a higher risk for postoperative delirium [14]. As shown in this study AChE increased within the first 3 days after surgery and BChE decreased. We do know from previous studies that serum cholinesterase (BChE) is the major enzyme hydrolysing AChE in the blood. The role of this enzyme during inflammation has not been fully understood yet. However, studies show that the reduction of BChE activity could early indicate the onset on the systemic inflammatory response. Therefore, BChE could decline due to the inflammatory response in the neurosystem after surgery.

Studies with an interventional approach using cholinesterase inhibitors such as physostigmine were performed. Dawson et al. proposed the use in anticholinergic delirium that did not respond to non-pharmacological delirium management [16]. In contrast, Jackson et al. assumed that simple enhancement of cholinergic neurotransmission may not be sufficient enough to treat delirium [23]. Most studies which were previously performed did not include patients undergoing cardiac surgery. There are various studies with patients in general surgery or on the internal ICU.

Potentially, there is an interaction of AChE and BChE and the use of a cardiopulmonary bypass. A different medication is used for those patients as well. Furthermore, patients after cardiac surgery are critically ill and do need transfusion of blood products. Until now no studies were performed which evaluated AChE and BChE and possible interactions of blood products. A recent study with patients undergoing venoarterial ECMO therapy after cardiac surgery however revealed BChE as a strong predictor of all-cause and cardiovascular mortality [19].

Another fact to mention is the different concentration of AChE at the perisynaptic space and the blood. Although previous studies show that the blood concentration is strongly related to the rate of delirium and a method for testing was developed, the pathophysiology is not clearly understood yet.

The latest systematic review about published RCTs evaluated the use of acetylcholinesterase inhibitors for delirium treatment. No efficacy for the prevention or management of delirium could be found [17].

Incidence of delirium measured with CAM-ICU was much lower than with Nu-DESC. Possible reasons might be more subjective evaluation of patients with Nu-DESC. Patients just need two points out of ten to be marked positive for delirium; that means already two items can be judged as low remarkable. Patients after cardiac surgery might easily be scored with positive items due to long duration of surgery. In general, those patients are more often very critically ill which also supports this statement. Another aspect might however also be that delirium assessment with Nu-DESC might identify patients in a prodromal stage of delirium. Furthermore, both methods have a different approach. Nu-DESC is a test with external assessment, *CAM-ICU* needs a reaction and interaction of the patient [21, 24].

## Conclusions

In conclusion, we could reproduce results regarding delirium as previously shown. Delirium is a serious complex of various symptoms, which need to be recognized early to initiate a correct and effective treatment. In this analysis, no difference of AChE and BChE in cardiosurgical patients with or without postoperative delirium could be found.

Further studies are needed to evaluate a possible connection of delirium and the cholinergic transmitter system. Studies which investigate the pathophysiology of the cholinergic system are essential. Furthermore, a possible association of cardiosurgical patients on the ICU and the cholinergic transmitter system should be examined. Studies measuring acetylcholinesterase and butyrylcholinesterase in surgical patients should include preoperative values and need to be continued during surgery and postoperatively (probably over more than three days after surgery). Not to mention the fact that the use of anticholingeric medication should be handled with care. Furthermore, the measurement should be performed more than twice a day and probably even at night. Due to the fact that mostly patients after cardiac surgery develop delirium, and due to the negative impact on clinical outcomes, more studies with this patient population should be conducted in the future.

## **Abbreviations**

AChE: Acetylcholinesterase
AMT: Abbreviated mental test
ASA-PS: American Society of Anesthesiologists–Physical Status
BChE: Butyrylcholinesterase
BIS: Bispectral index
CAM-ICU: Confusion Assessment Method for the Intensive Care Unit
CI: Confidence interval
CK: Creatine kinase
CK-MB: Creatine kinase, myocardial subtype
CRP: C-reactive protein
DSM-5: Diagnostic and Statistical Manual of Mental Disorders, Fifth Edition
ECMO: Extra-corporal-membrane-oxygenation
HADS-D: Hospital Anxiety and Depression Scale—German version
ICU: Intensive care unit
IL-1/IL-6: Interleukin-1/-6
LVEF: Left ventricular ejection fraction
M: Mean
NIRS: Near-infrared spectroscopy
NRS: Numeric rating scale
Nu-DESC: Nursing Delirium Screening Scale
p: *p* value
POD: Postoperative delirium
PSQ: Pain Sensitivity Questionnaire
RASS-Score: Richmond Agitation Sedation Score
RCT: Randomized controlled trial
SD: Standard deviation
SF-12: Questionnaire for Quality of life
TEE: Transesophageal echocardiography
